# Is obesity a risk factor for low back pain? An example of using the evidence to answer a clinical question

**DOI:** 10.1186/1746-1340-13-2

**Published:** 2005-04-11

**Authors:** Timothy A Mirtz, Leon Greene

**Affiliations:** 1 University of Kansas, Department of Health, Sport, and Exercise Science. Lawrence, Kansas, USA

**Keywords:** Low back pain, obesity, association, risk factor, evidence-based practice

## Abstract

**Background:**

Obesity as a causal factor for low back pain has been controversial with no definitive answer to this date. The objective of this study was to determine whether obesity is associated with low back pain. In addition this paper aims to provide a step-by-step guide for chiropractors and osteopaths on how to ask and answer a clinical question using the literature.

**Methods:**

A literature review using the MEDLINE search engine using the keywords "obesity", "low back pain", "body mass index" "BMI" and "osteoarthritis" from years 1990 to 2004 was utilised. The method employed is similar to that utilised by evidence-based practice advocates.

**Results:**

The available data at this time is controversial with no clear-cut evidence connecting low back pain with obesity.

**Conclusion:**

There is a lack of a clear dose-response relationship between body mass index (BMI) and low back pain. Further, studies on the relationship between obesity and related lumbar osteoarthritis, knee pain, and disc herniation are also problematic.There is little doubt that future studies with controlled variables are needed to determine the existence of an unambiguous link, if any.

## Introduction

Obesity is a problem of epidemic proportion [1,2]. Despite record rates of non-physician supervised dieting and the availability of numerous weight loss programs, the problem is not abating [3]. Complicating this, is that most primary care physicians do not treat obesity, citing a lack of time, resources, insurance reimbursement, and knowledge of effective interventions as significant barriers [4]. Musculoskeletal disorders including low back pain (LBP) represent a considerable public health problem and a common diagnosis creating absenteeism and the need for disability pensions [5]. It is estimated that about 80% of the United States and Canadian population will experience LBP during adulthood [6]. Most low back pain is self-limiting and will ultimately resolve in two weeks (50% of those affected) to six weeks (90% of those affected), however it remains an intriguing clinical problem [6].

It is widely noted that the economic cost of obesity and its related disorders are staggering, with lifestyle related conditions such as diabetes mellitus and coronary heart disease placing a large economic burden on the health care system [1-4]. However, low back pain also has a significant socioeconomic impact. Cost estimates range from US$20 billion to $50 billion annually, with 10% of the patients accounting for 85 to 90% of the costs [6]. In Australia, Walker et al estimated the cost of low back pain in 2001 alone to be AUD$9.17 billion [7].

One question, which arises from the discussion concerning obesity, is whether obesity is a risk factor for low back pain. "Buckwalter et al contended that a number of medical conditions including obesity, along with diabetes and hypertension, may influence the pathophysiology of diseases of the tendons and ligaments during the process of aging thus potentially leading to low back pain [8]. Along with low back pain, the conventional wisdom is that overweight persons are at risk of osteoarthritis in weight-bearing joints such as the knee, the hips, and feet [9].

To date, literature reviews have given conflicting views based on the available data and method of data retrieval. The purpose of this review is to establish, from recent research, if there is a causal link between obesity and the affliction of low back pain. A secondary purpose of this review is to present the concepts of evidence-based practice to aid the chiropractor or osteopath in looking for health-related evidence for their patients who present with obesity.

## Methods

A MEDLINE search, from the National Library of Medicine, was used to ascertain pertinent articles between the years 1990 and 2004. The use of keywords "obesity", "body mass index", "BMI" and "low back pain" was used to obtain relevant studies. The references of papers retrieved were also reviewed, as were key texts and references.

This section is devoted to presenting the concepts of evidence-based practice (EBP) to demonstrate to the reader the discovery process for finding a possible link between obesity and low back pain. The EBP method used is shown in Table 1.

**Table 1:** Steps to asking the answerable question using EBP principles

Step 1: Asking an answerable question
Step 2: Selecting an evidence resource
Step 3: Executing the search strategy
Step 4: Examining the evidence summary
Step 5: Application of the evidence

The first step in this process is "asking an answerable question." In this paper we assume a patient has asked whether being overweight can cause low back pain. Construction of an appropriate answerable question would possibly be "Does an increased BMI cause low back pain?" "Does being overweight create osteoarthritis?" In this way questions can be constructed to allow the practitioner to effectively answer a clinical concern.

Once the answerable question has been constructed the next task is to find an adequate resource. Internet access to the U.S. National Library of Medicine's MEDLINE or PUBMED, these database systems are considered by many experts to be the most up to date data source on medically related topics. The next step is to determine keywords to place in the search engine. From the answerable question(s) it can be appreciated that the initial keywords will be "low back pain", "BMI" and "osteoarthritis." This search constitutes the third step. In initiating the search, one should look for the search engines "limits" area. In this area can one designate an age group (ex: 45 to 60 years), date span of the literature search, (ex: 1998 to 2003), and to select either English language or articles in foreign languages.

Once the search has been completed, the articles, which may answer the question, are isolated and can be read. Step four involves collating the evidence to answer the question. In searches one may find answers that were not known to exist, and information that may challenge an already preconceived notion. The evidence summary should list the main author and year the paper was published. This is in order to retrieve the data should anyone wish to examine the source. As an example of an evidence summary see Table 2.

**Table 2:** Recent evidence: Obesity and low back pain (chronological order)

**Author, Year (Ref)**	**N**	**BMI**	**LBP**	**Association**
Melissas, 2003 [14]	50	>40	58%	direct
Bener, 2003 [10]	802	(26.4 males/ 27.8 females)	56.1% males	
			73.8% females	moderate
Tsuritani, 2002 [16]	709	--	40.3%	none
Bowerman, 2001 [4]	252	--	29.2%	none
Kostova, 2001 [12]	898	--	--	increased risk
Bayramoglu, 2001 [15]	25	--	--	direct
Mortimer, 2001 [13]	475	30 (43.6%)		
		31–40 (28.8%)		
		40+ (1.3%)	**--**	increased risk
Han, 1997 [11] 7018 women	5887 men NR	--		females increased risk

N = number; BMI = body mass index; LBP = low back pain; NR = not reported

It is at this time that the clinician is ready for the final step of applying the evidence. In our example clinical data from the experimental literature may or may not indicate that there is a link between overweight and low back pain.

## Results

The literature search into obesity and joint pain revealed several studies pertinent to the debate. Table 2 reveals an overview of the studies selected. Several studies [4,10-13] had large populations to draw from yet the data from these studies were not in agreement as to a cause or association. In fact, only two studies [14,15] found a direct association for obesity as a risk factor while two [4,16] studies found no association. Several of the studies reviewed were unable to clarify BMI to the satisfaction of the authors.

## Discussion

Interest in the association between obesity and low back pain has piqued researchers interest for many years. Intuitively, a burgeoning waistline and an increased lordotic lumbar spine led researchers to conclude that overweight people would be more prone to low back pain. Historically, Kellgren and Lawrence (1958) found that that the prevalence of disk degeneration with obesity was not significant [17]. However, it was not until the mid-1970's when several studies observed a possible association. Obesity was found to increase the prevalence of disk degeneration significantly in a study by Magora and Schwartz in 1976 [18]. Barton et al (1976), in a review of 144 cases, found that 70% of those who complained of low back pain had been classified as being overweight [19]. This basic research appeared to conclude what was already intuitively thought about low back pain and increased weight.

### Body mass index

Before an in-depth discussion of low back pain and obesity can ensue, the concept of Body Mass Index (BMI) needs to be discussed. BMI is a measure of fatness and is calculated by dividing the patient's weight in kilograms by height in metres squared kg/M^2 ^[20]. It is widely accepted, easily measured, and predicts morbidity and mortality in many populations [15]. Obesity is generally defined as a BMI of 30 kg/m2 and higher [20,21]. Overweight is defined as a BMI between 25 and 30 kg/m2 [19,20]. Overweight tends to be more common in men with obesity being more prevalent among women [21]. When body weight is increased 20% above average, mortality rises to 20% for men and 10% for women [22]. (Table 3) Overweight individuals demand more from their cardio-respiratory and musculoskeletal systems [22]. It is known that more than 50% of adult Americans have a BMI equal to or greater than 25 [23]. Although there are certain limitations to BMI i.e. large muscular athletes who are in good cardiovascular shape, the rationale behind these numbers is that, across large population groups, there is an increased prevalence of certain diseases in people with a BMI over 25, and a much greater risk of disease and death in those with a BMI over 30 [4]. Being overweight or obese substantially raises the risk of developing hypertension, coronary heart disease, type 2 diabetes, stroke, gallbladder disease, sleep apnea and other respiratory problems, prostate and colon cancers [4,23]. Yet, the evidence to date linking it to low back pain is not as clear cut as it is with the previously stated pathologies.

**Table 3:** Clinically relevant differentiation between obesity and overweight

**Overweight**	**Obesity**
BMI of 25.0 to 29.9 kg/m^2^	BMI greater than 30 kg/m^2^

### BMI calculation without benefit of BMI charts

Body Mass Index (BMI) charts and hand held scales are available for individual clinician use. It is, however, unknown to what degree chiropractors or osteopaths use such tools. The following section is designed to aid the clinician with calculating BMI without benefit of chart or hand held scales.

As noted earlier, BMI is calculated as weight in kilograms divided by height in square metres [20,24]. This method is often too difficult to calculate for most people. A simpler method for those using the imperial system of measures is to take body weight in pounds × 703/height in inches squared.

For example, a person weighing 150 pounds at 6 foot tall would correspond to a BMI of 20.3. TABLE 4

**Table 4:** Calculation of BMI

150 × 703 = 105450 divided by 72 inches (6 foot) squared.
105450 divided by 5184 (72 × 72) = 20.3 BMI.

### Additional research findings

Leboeuf-Yde concluded from a review of the literature that due to lack of evidence, body weight should be considered a possible weak risk indicator and suggested that there is insufficient data to assess if it is a true cause of LBP [25]. Kostova found that in men over 40, overweight, obesity and number of pack years of smoking, estimated by duration of smoking and daily cigarette consumption (more than 20 years and more than 20 cigarettes per day), increased the risk of developing back disorders [12].

Despite these two studies, Garzillo et al and Leboeuf-Yde et al have given conflicting opinions [26,27]. Garzillo's review of the data revealed a possible association between obesity and low back pain only in the upper quintile of obesity, and no evidence of a temporal relationship between weight change and changes in low back pain [26]. Leboeuf-Yde concluded from a twin study that obesity is modestly positively associated with low back pain, in particular with chronic or recurrent low back pain [27].

What appears to be a main concern in linking obesity as a causal factor for low back pain is the numerous variables encountered in these subjects. For example, it is hypothesized that overweight adult females may have negative self-concepts and body images compounded by chronic low back pain and obesity, these may be confounding factors [28]. Other variables such as less activity and/or muscular weakness leading to obesity are also possible considerations.

### Obesity and low back pain-related conditions

Not only is there controversy in obesity and low back pain, but there exists conflicting views of obesity and low back pain-related conditions such as spondylosis, decreased physical activities and discal herniation. The studies demonstrating a positive association are many. O'Neil *et al *noted that increasing BMI is associated with more frequent findings of osteophytes (bone spurs) at both the thoracic and lumbar spines [29]. The correlation of osteophytes and increased BMI is highest at the thoracic level [29]. Biering-Strenson *et al *noted absolute weight and BMI are significantly higher in persons 60 years of age with spondylosis [30]. Both men and women with BMI of 30 kg/m2 or higher were twice as likely to have difficulties in performing a range of basic daily physical activities [30]. Compared with women with BMI lower than 25 kg/m2, those with BMI of 30 kg/m2 or higher were 1.5 times more likely to have symptoms of intervertebral disk herniation [31].

Conversely, Luoma *et al *concluded that disc degeneration is not related to body height, overweight, smoking, or the frequency of physical activity [32]. In addition, studies by Riihimaki, Symmons, and Kang have shown no association between BMI and low back related problems [33-35].

Confounding the data is that the mechanism by which excess body weight causes osteoarthritis is poorly understood [9]. It is believed that contributions from both local increased force across the joint and systemic factors play a role [9]. A discriminating factor between fit and unfit patients with back pain may be the fact that fit persons more frequently are still employed, and as such may be involved more in physical activity [36]. Table 5 indicates where the research currently exists for the link between low back pain and obesity along with obesity and osteoarthritis.

**Table 5:** BMI-related risk of osteoarthritis and low back pain

If your BMI is	then your risk based solely on BMI
<25	minimal
25 – <27	minimal
27 – <30	minimal
30 – <35	moderate
35 – <40	moderate
>40	moderate to high

We conclude, based on the available evidence to date, that those individuals with a BMI of under 30 are at a minimal risk of developing low back pain while those persons whose BMI increases to over 30 are a moderate risk of developing low back pain. We also suggest, based on the findings of the Melissas study [14] of those patients who relieved their low back pain symptoms after obesity surgery, that patients with a BMI of greater than 40 are at a high risk of developing low back pain. Albeit controversial, Table 5 may lead to a further refinement of risk of osteoarthritis and low back pain based solely on BMI.

### Limitations of obesity as a risk factor for low back pain

A significant difficulty in ascertaining cause and effect between obesity and low back pain is undoubtedly the term "low back pain" itself. Low back pain is a symptom not a diagnosis. A specific diagnosis, instead of the generalized form of "low back pain" may help separate out the association between LBP and obesity.

The Agency for Health Care Policy and Research (AHCPR) in their 1995 *Acute Low Back Problems in Adults *noted common diagnoses used to explain back problems [37] (Table 6). Given these possible diagnoses one can readily appreciate the dilemma in attempting to link obesity with its specificity in measurement to a broad symptom such as low back pain.

**Table 6:** Common diagnoses used to explain back symptoms

Annular tear	Adult spondylolysis	Myofascitis
Fibromyalgia	Disc syndrome	Strain
Spondylosis	Lumbar disc disease	Facet syndrome
Degenerative joint disease	Sprain	Spinal OA
Disc derangement/disruption		Dislocation
	*Other potential causes of low back pain symptomology	
Failed Back Surgery Syndrome*		Osteoporosis*
Urinary tract infection*		Compression fracture*
Somato-visceral mimicry syndrome*		
Organic pathology (tumor, rheumatoid, endometriosis, arthritic disorders)*		
Leg length inequity*		Sacro-iliac dysfunction*
Hip disorder*		
	**Disagreement in research as cause of low back symptomology	
Morbid obesity?**		

OA = osteoarthritis

Another problem is the hypothesis that a person who suffers with continuing bouts of low back pain may be predisposed, due to inactivity or inability to exercise, to gain weight thus increasing their BMI. This hypothesis to our knowledge, has yet to be fully discussed and investigated.

## Conclusion

The data for a link between obesity and low back pain appears to be controversial. Yet, this does not adequately address the appropriate therapeutic approach to the obese patient with low back pain. The studies chosen for this review fail to document a definitive causal link between obesity and low back pain. Further research and epidemiologic data is needed to continue the search for a definitive answer.

## Competing interests

The author(s) declare that they have no competing interests.
